# To complain or not to complain: Management responses as a moderator in the relationship between workplace incivility and workplace outcomes among Australia and singaporean targets

**DOI:** 10.1016/j.heliyon.2023.e21363

**Published:** 2023-10-21

**Authors:** Jennifer (M.I) Loh, Md Irfanuzzaman Khan, Md Jakir Hasan Talukder

**Affiliations:** a Faculty of Business, Government and Law, Canberra Business School, University of Canberra, Bruce Campus, Canberra, ACT, Australia; b Faculty of Business, Government and Law, Canberra Business School, University of Canberra, Bruce Campus, Canberra, ACT, Australia; c Department of Marketing, Jagannath University, Bangladesh

**Keywords:** Workplace incivility, Formal complaints, National culture, Work satisfaction, Work withdrawal

## Abstract

Workplace incivility is a challenging global occupational risk that is frequently considered trivial by managers and organizations. Often, complaints from targets are ignored; when this occurs, complaints can quickly escalate into formal grievances that cost businesses millions of dollars. While existing studies have uncovered cultural and gendered differences in how targets and organizations respond to workplace incivility, few cross-cultural studies have empirically examined how targets and organizations react to formal complaints. This study responds to this gap by using selective incivility, the transactional stress model, and national/cultural theories to conduct a multifaceted analysis of the underlying mechanisms responsible for targets’ organizational outcomes. Specifically, we tested a moderated model with 303 Australian (152 males and 151 females) and 304 Singaporean (154 males and 150 females) employees working in multinational organizations to determine whether the degree to which organizations took incivility complaints seriously moderated the organizational outcomes of work withdrawal and work satisfaction. Overall, the results indicated that, compared to Singaporean employees and Australian female employees, Australian male employees were less tolerant of being mistreated and continued to experience heightened job dissatisfaction and withdrawal even when their complaints were taken seriously by their organization. These results suggest that complex gendered and cultural differences influence the impact of incivility complaints on work-related outcomes.

## 1 Introduction

Workplace incivility is a psychosocial hazard that many business leaders find difficult to address. It has been conceptualized as “low-intensity, disrespectful or rude deviant workplace behavior with ambiguous intent to harm the target and is in violation of workplace norms for mutual respect” ([1], p.457); put differently, workplace incivility refers to thoughtless and rude deeds that ignore or violate conventional norms of respectful and ethical behavior in the workplace [2]. Notably, these actions are subtle, passive, and indirect [1]. The intent to cause harm is often vague, making the true intention of the perpetrator difficult to decipher [3]. In addition, this uncertainty can also be attributed to varying perceptions of what comprises a “rude” action; for example, someone may not say “thank you” because they are forgetful rather than because they are trying to be rude—nevertheless, the person expecting a “thank you” may still interpret the failure to say it as rude if they are hypersensitive. This ambiguity makes it difficult to address workplace incivility. Meanwhile, others have further suggested that workplace incivility can be rooted in carelessness [4]; for example, it has been conceptualized as “subtle rude or disrespectful behaviors that demonstrate lack of regard for others”, such as dismissive gestures, hostile stares, the silent treatment, or not saying “please” or “thank you [4, p. 702].”

### 1.1 Literature review

Workplace incivility can harm both employees and employers. Specifically, ongoing animosity among colleagues can lead to significant job dissatisfaction, psychological stress, physical health problems, and high levels of employee withdrawal and exit [[5], [6], [7], [8], [9], [10]]. For example, a sample of Nigerian health workers (n = 447) found that work–family conflict (WFC), family–work conflict, supervisor incivility, and coworker incivility contributed positively to psychological distress, especially among women [11]. Similarly, another study [12] using data collected from frontline workers in 3-star hotels found that favoritism and supervisor incivility positively impacted employee cynicism and work withdrawal. These two studies provide important insights into the detrimental consequences (i.e., psychological distress, cynicism, and work withdrawal) of workplace incivility. Other scholars have proposed the need to include potential moderators, such as tolerance, in studies of workplace incivility to better explain its nonlinear relationship with negative outcomes. More specifically, tolerance of workplace incivility may moderate its relationship with organizational outcomes because tolerance has been found to impact employee cynicism, job search behavior, job performance, and intention to sabotage [[13], [14], [15], [16]].

Additionally, scholars have further found that incivility can trigger an escalating spiral of negative behaviors, where exchanges of seemingly small insults can deteriorate into vengeful aggression and possibly even violence [1]. According to researchers [5], the escalating spiral of the incivility model is consistent with research reporting a positive connection between toxic work environments and dysfunctional interpersonal relationships in the workplace. For example, researchers [8,17] showed that targets of incivility retaliated against negative behaviors by behaving negatively towards their perpetrators. Similarly, a recent longitudinal study of 1005 engineers in Sweden found that being a target or witness of workplace incivility increased the risk of being bullied in the future [18]. These studies suggest that exposure to workplace incivility makes employees highly susceptible to being bullied in the future and can negatively impact employees and their workplace relationships [19].

Despite its serious consequences, workplace incivility is often ignored by business leaders [20,21]. Indeed, most employees who experience workplace misconduct do not formally report negative experiences [22,23]. This mistrust among victims is highly problematic because it can lead to a culture of silence in which negative workplace misconduct goes unchecked, ultimately damaging company integrity over time. Therefore, there is a need to address the question of organizational responses to targets’ complaints in a more direct and critical manner.

While the antecedents, prevalence, and effects of workplace incivility have been researched [5,9,[24], [25], [26], [27], [28]], few studies have empirically measured target responses [8,29] or how organizations handle targets’ formal complaints. Moreover, emerging research has found significant variations in responses to workplace incivility based on cultural and gendered differences, with individuals from high power distance cultures (e.g., Singapore) demonstrating more accepting attitudes towards workplace misconduct compared to individuals from low power distance cultures (e.g., Australia [29,30]). For instance, several scholars [29,31] have found that women or targets from high power distance cultures (e.g., Singapore) were more tolerant of workplace mistreatment than men or victims from low power distance cultures (e.g., Australia). According to Abubakar et al. [14], tolerance of workplace incivility reflects an organizational climate that allows uncivil behaviors, evident in a lack of appropriate management responses to workplace incivility [14]. Therefore, the climate of an organization plays an important distinguishing role in determining what constitute workplace incivility versus what constitute tolerance to workplace incivility.

Although responses to workplace incivility seem to differ based on culture and gender, few studies have examined workplace incivility from a cross-cultural perspective. Additionally, limited research has been done on how formal complaints are handled by multinational organizations and how targets from different cultures and/or genders react when their formal complaints are handled inappropriately. Therefore, this study aimed to address these gaps in the workplace incivility literature by conducting a cross-cultural study that empirically investigated the experiences of workplace incivility of Australian and Singaporean targets. Further, given the limited understanding of the role organizations play in the relationship between workplace incivility and outcomes, this study conducted a multifaceted multilayered analysis (i.e., at the individual, organizational, and societal levels) to determine whether organizational responses to targets’ complaints moderated the relationship between workplace incivility and organizational outcomes (specifically: work satisfaction and withdrawal) between Australian and Singaporean employees.

## 2 Contributions to the literature

This study contributes to workplace incivility literature in three main ways. First, it analyses workplace incivility from an ecological perspective rather than an individual one, thereby expanding research beyond the individual level. Here, an ecological approach refers to a work environment that is “conceived topologically as a nested arrangement of concentric structures, each of which is contained within the next. These structures are referred to as micro-, meso-, exo-, and macrosystems …” ([32], p. 22). In this case, the mesosystem and macrosystem are represented by organizational responses and national/cultural groups, respectively. In other words, we are interested in both the interpersonal interactions between victims and targets and the interplay between organizations and individuals across different countries. This requires a multifaceted analysis that incorporates more than one theory. Therefore, we incorporated selective incivility theory, the transactional stress model, and national/cultural theories to provide an integrated explanation of the reciprocal ways in which individuals, organizations, and national/cultural groups interact to influence targets’ outcomes when organizations ignore their formal complaints.

Second, the cross-cultural nature of this study responds to the call for more research with samples comprising targets of workplace incivility beyond Western cultures [33]. This research is needed because individuals from different cultures have different values, norms, beliefs, and/or reactions towards workplace misconduct. Additionally, the growing likelihood that employees will work in increasingly global and diverse organizations further evidences the need to conduct analyses of workplace incivility across different cultures. Finally, the findings from this cross-cultural study will contribute to practice by offering insights that may enable researchers, human resources, and management leaders to develop conflict-resolution strategies that are responsive to victims’ cultures and genders and thus to break away from one-size-fits-all strategies.

### 2.1 Theoretical framework

#### 2.1.1 Selective incivility theory

The selective incivility theory is a multilevel theory that describes how subtle and ambiguous acts of rudeness may function as covert manifestations of bias against devalued, stigmatized, or marginalized people in organizations. Importantly, this theory provides a multilevel analysis of “modern” biases and discriminations in organizations that include the individual, organization, and society. This is important because it provides a powerful multilevel framework that delineates how incivility operates through the subtle layers of multiple realities [34,35]. Employees of different cultures and genders experience these realities differently. Importantly, individuals’ social, cultural, and gender identities overlap to create different types of discrimination or disadvantages, such as sexism, racism, classism, and heterosexism [36,37], which likely lead to differential organizational responses to their complaints. This theory is therefore especially useful in developing a nuanced explanation for the existence of different treatments and responses (e.g., how organizations respond to formal complaints) between male and female Australian and Singaporean employees working in multinational organizations.

At the individual level, selective incivility centers on individual differences, such as negative affect/emotionality [38,39]; differences in core self-evaluation and self-esteem [40,41]; and cognitive factors, such as social categorization and stereotyping [42,43]. At the organizational level, the physical aspects of the work environment, supportive leadership, extent of social/emotional support at work, intra-organizational and socio-cultural norms, and policies against discrimination may shape selective incivility [[44], [45], [46], [47]]. In other words, selective incivility provides an ecological and multilayered explanation of workplace incivility [34,35]. Finally, at the societal level, scholars haveproposed that a tradition of discrimination, asymmetrical intergroup power relations, and social and gender role differences likely influence selective incivility [34,[48], [49], [50], [51]]. For example, researchers have found evidence of subtle racism in UK workplaces, where racialized professionals appear to be the main targets of selective incivility [52].

However, few studies have considered all three levels of workplace incivility. Therefore, this study addresses this gap in the workplace incivility literature by conducting a cross-cultural study to empirically investigate the experiences of workplace incivility among Australian and Singaporean victims. This study also investigated the potential underlying mechanisms to determine their influence on the victims’ work outcomes.

It is important to note that while incivility may first appear to be less important than workplace bullying, it is still a highly stressful event for targets—it depletes mental resources, leaving victims highly defensive, aggressive, and irrational [29]. Crucially, stress is not only relevant to highly stressful events or incidents; regular exposure to minor stressful events can also cause great strain over time [53]. Workplace incivility is one such “daily hassle” [54].

#### 2.1.2 Transactional model of stress and coping

The transactional model of stress and coping contends that psychological stress occurs in the presence of a “relationship between the person and the environment that is appraised by the person as taxing or exceeding his or her resources and endangering his or her wellbeing [53, p. 19]”. In other words, a person's capacity to cope with and adjust to challenges (e.g., workplace incivility) is a consequence of transactions (or interactions) that occur between a person and their environment. More importantly, this model proposes two states of appraisal; namely: (1) primary appraisal and (2) secondary appraisal-coping. The primary appraisal state refers to an individual's judgment of an event or incident as stressful or benign [53]. If the situation is benign, then no specific instrumental action is required by the individual, whereas a stressful situation requires specific actions. Threatening situations, such as workplace incivility, are likely to elicit a response (i.e., a secondary appraisal) in which the target evaluates whether he/she has sufficient resources to cope [53]. If the target perceives the event as stressful and does not have the resources to cope with it, then he/she will experience negative stress and start engaging in coping strategies, such as problem- or emotion-focused coping. With problem-focused coping, individuals attempt to change negative situations by seeking professional support [55]. In contrast, emotion-focused strategies involve victims changing their emotional response to a situation instead of trying to solve the problem itself through strategies such as disclaiming (denial), escape-avoidance, accepting responsibility or blame, exercising self-control, and engaging in positive reappraisal [55].

#### 2.1.3 Impact of workplace incivility on work withdrawal and work satisfaction

As discussed above, targets of workplace incivility live in highly stressful environments characterized by intensifying spirals of negative “tit for tat” behaviors. Indeed, as noted above, researchers have found that workplace incivility is a heightened stressor that exposes targets to psychological distress, such as emotional exhaustion, burnout, and low intrinsic motivation [7,29,54,56]. In terms of adverse work outcomes, workplace incivility has been found to be positively associated with reduced job commitment/engagement/performance, absenteeism, turnover, job dissatisfaction, and work withdrawal [6,26,[57], [58], [59], [60]].

Work withdrawal refers to negative behaviors engaged in by dissatisfied employees to avoid performing specific work tasks [[61], [62], [63]]. This includes leaving work early, taking longer lunches or breaks, being late to work, and letting others do their job. Similarly, researchers have found that workplace incivility was strongly negatively related to work satisfaction [30,64,65]. In this study, work satisfaction is defined as employees’ overall global satisfaction with their job [66]. Finally, a literature review also found a negative association between work withdrawal and job satisfaction [67,68]. Based on these empirical studies, we propose the following hypotheses:

H1 Workplace incivility is negatively related to work satisfaction.

H2 Workplace incivility is positively related to work withdrawal.

H3 Work satisfaction is negatively related to work withdrawal.

Although the transactional model of stress and coping provides important insights into why workplace incivility is a stressor [53], targets of workplace incivility may not always know the sources of their stress—this uncertainty is further compounded by the potential ambiguity of perpetrators’ intentions, as discussed above. For example, if your supervisor ignores your raised hand or glares at you in a meeting, is he/she rude? Should you confront them or ignore them? What we do know is that no matter how subtle or minor workplace incivility seems, incivility is likely to erode respectful norms in the workplace and eventually cultivate a culture where interpersonal rudeness spreads like wildfire throughout the organization [1,69,70]. This form of interpersonal conflict can become an aggressive problem that is difficult for management to resolve. Importantly, targets of workplace incivility seldom complain about their negative experiences because the ambiguity surrounding workplace incivility makes it difficult to identify. Moreover, the lack of organizational policies to appropriately tackle or ameliorate such ambiguous situations makes victims reluctant to come forward [[71], [72], [73]]. Further research in this area is required.

#### 2.1.4 Organizational responses to victim complaints as organizational injustice

Research suggests that perceptions of fairness and justice in organizations can impact workplace incivility. Organizations often fail to seriously consider workplace incivility, which, in turn, affects employee commitment and may result in further uncivil employee behavior [3]. For instance, employees may retaliate against incivility by acting aggressively against their organization or supervisor when they perceive a lack of organizational justice [74]. Similarly, when employees perceive that they have not been treated fairly, they are likely to blame and retaliate against their supervisors [75,76]—some even take drastic actions with tragic consequences, such as suicide [77].

A study has also found that employees who complained felt that they had not been treated fairly or equitably [78]. Employees’ complaints in the workplace signify that they are unhappy and suffering, which can intensify their sense of organizational injustice, leading to work withdrawal and reduced work satisfaction. Accordingly, more research should be conducted in this area to determine whether serious organizational responses to workplace incivility improve work satisfaction. Therefore, we proposed that the degree to which organizations take incivility complaints seriously moderates the relationship between workplace incivility and outcomes (e.g., work satisfaction and work withdrawal).

H4 The level of seriousness with which organizations respond to workplace incivility complaints moderates the relationship between workplace incivility and work satisfaction, such that the negative relationship between workplace incivility and work satisfaction is stronger when organizations take complaints less seriously.

H5 The level of seriousness with which organizations respond to towards workplace incivility complaints moderates the relationship between workplace incivility and work withdrawal, such that the positive relationship between workplace incivility and work withdrawal is stronger when organizations take complaints less seriously.

#### 2.1.5 National culture

National/cultural norms, beliefs, and values not only influence the manifestation of selective incivility but also determine how organizations respond to complaints of workplace incivility. In this study, national culture/ethnicity is conceptualized as a category of people who identify with each other based on their cultural and national backgrounds, including their ancestry, regional culture, language, and nationality [79]. Hofstede noted that culture affects beliefs, values, and ways of doing things [[80], [81]]. For example, in high power distance cultures, such as China and Singapore, where hierarchical ways of thinking and being are normal, there is an expectation that subordinates will adhere and defer to their superiors’ authority. This suggests that power distance—that is, “the extent to which the members of a society accept that power in institutions and organizations is distributed unequally” [81, p. 347]—is likely to affect not just workplace incivility but also perceptions of inequality and how workplace misconduct is handled.

According to Hofstede, cultures differ in their levels of power distance, and those with high power distance (e.g., Malaysia, Singapore, and China) justify societal or ingroup inequalities, whereas those with low power distance (e.g., Australia and New Zealand) are more concerned with maintaining equality [82]. For example, in collectivist cultures, such as China and Singapore, “inequality is seen as the basis of societal order [83, p. 97]; therefore, employees tend to accept and tolerate power differences, prefer non-confrontational forms of conflict resolution [83], and defer to their superiors’ decision without question [84]. In other words, individuals and organizations in high power distance cultures, such as Singapore, tend to treat workplace misconduct less seriously and are more likely to tolerate such workplace misconduct compared to individuals in low power distance cultures. Therefore, employees in individualist and low power distance cultures may be less tolerant of hierarchical inequalities or workplace mistreatment than individuals from collectivist cultures (e.g., Singapore) [[85], [86], [87], [88]].

Based on the above, we argue that employees from countries with higher power distances will be better accustomed to accepting and adapting to workplace incivility because they perceive it as a natural element of the hierarchical organizational structure. In contrast, employees from countries with lower power distances (e.g., Australia) may be more negatively affected by workplace incivility [29]. This is because employees from low power distance countries expect fair treatment from colleagues and supervisors [89]. Owing to this inherent expectation of equal treatment and respect at work, employees from low power distance countries are more likely to be intolerant of workplace incivility [90] as they perceive such incivility as a serious breach of respectful and equitable treatment. Therefore, we proposed the following hypothesis:

H6 Australian participants are more adversely impacted by workplace incivility in terms of work withdrawal and work satisfaction than Singaporean participants, even when complaints are taken seriously by their organizations.

#### 2.1.6 Differences in gender roles

The selective incivility theory posits that incivility is a modern form of discrimination against those who hold marginalized identities, including women, people of color, and older adults. Selective incivility “operates as a covert, interpersonal mechanism through which socio-structural discrimination and mistreatment manifest to maintain status, power and privileges for certain groups over others [33, p. 257]”. Indeed, past research has found an increased risk of workplace incivility for minorities and people from high power distance cultures, as well as differential responses and reactions from victims [29,30,47,91,[92], [93]]. For instance, a study in Australia found that women who experienced workplace incivility were more hesitant than men to “rock the boat” and less likely to withdraw from their work despite being treated uncivilly. Victimized women also tend to protect themselves by “silencing themselves” [93, p. 176]; they “hide their conflict and ‘put up’ with life injustices” [94].

This research aligns with Hofstede's delineation of masculine and feminine cultures [82]. Singapore scored 48 on the masculinity scale, while Australia scored 61, suggesting that Singapore is more feminine than Australia. Importantly, in masculine cultures, such as Australia, men are generally expected to be assertive, tough, and focused on material success, while women are expected to be modest, tender, and concerned with quality of life [82]. Consequently, Australian men are expected to behave in a macho way to sustain their sense of masculinity and self-esteem, especially in the face of conflict. This suggests that Australian men are less likely to tolerate workplace incivility than Australian women, Singaporean men, and Singaporean women. Therefore, we derived the following hypothesis:

H7 Australian male participants are more adversely affected by workplace incivility in terms of work withdrawal and work satisfaction than Australian female participants and Singaporean participants, even when complaints are taken seriously by their organizations.

**Fig. 1:**
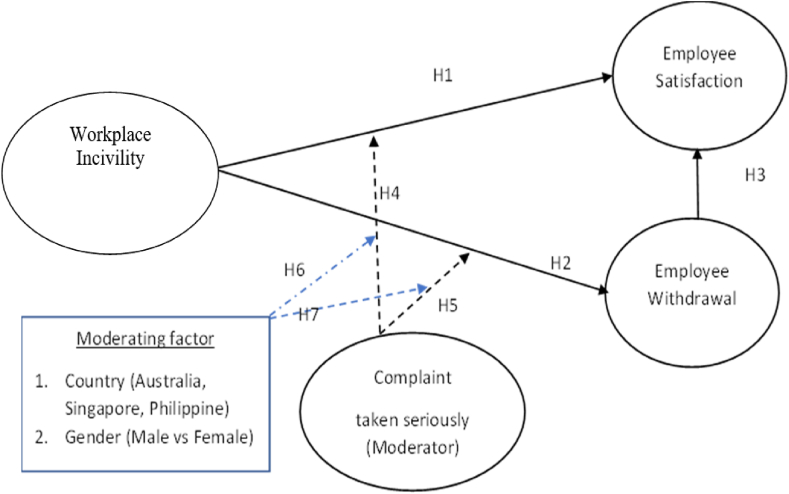
Conceptual framework.

The hypothesized relationships are conceptualized in Fig. 1 below.

## 3 Materials and methods

### 3.1 Participants

The participants were recruited through a Qualtrics panel. Qualtrics is a technology platform used to recruit targeted samples based on predetermined criteria (e.g., age, ethnicity, nationality, and lifestyle), allowing researchers to target the exact population they desire [95]. Within this platform, participants were recruited through various social media sites; notably, the platform enables researchers to quickly access a pool of potential participants within its ecosystem. The participant pool of this study encompasses various service industries, including banking, professional services, administrative roles, sales, and legal services. Participants in these roles were selected based on the following criteria: 18 years or older; comprehension of basic written English; and currently working as white-collar employees in managerial, professional, clerical, administrative, or sales roles.

This study included 607 participants. The participant pool was divided based on the study's two geographical locations: 303 participants were from Australia and 304 participants were from Singapore. Furthermore, each location-based group was categorized by sex, with 152 males and 151 females in the Australian group, and 154 males and 150 females in the Singaporean group. The mean age of the Australian participants was 44 years (SD = 12.6, ranging from 18 to 72) and the mean age of the Singaporean participants was 37 years (SD = 9.3, ranging from 18 to 67). Detailed demographic profiles of the participants are shown in Table 1.

**Table 1:** Demographical information.

Variables (sub-categories)	Australia		Singapore	
	Frequency	Percentage	Frequency	Percentage
**Gender**				
Female	151	50 %	150	49 %
Male	152	50 %	154	51 %
**Income**				
0 - $18,200	37	13 %	47	16 %
$18,201 - $37,000	65	22 %	83	27 %
$37, 001-$54,000	67	23 %	76	25 %
$54,001-$72,800	44	15 %	47	16 %
$72,801-$91,600	47	16 %	31	10 %
Above $91,601	32	11 %	19	6 %
**Marital Status**				
Married/Defacto	64	21 %	119	39 %
Single	204	67 %	177	58 %
Separated	4	1 %	4	1 %
Divorced	24	8 %	4	1 %
Widowed/Widower	7	2 %	0	0 %
Mean- Age	44		37	
Standard deviation	12.6		9.3	
Max-min	72–18		67–18	

### 3.2 Procedures

This study consisted of a quantitative survey distributed to white-collar workers in Australia and Singapore. Ethical approval was obtained from the University Human Research Ethics Committee (10230LOH). Participants were treated with the utmost respect. Participants were also (a) given clearly written instructions on the information letter, (b) advised that completion of the survey implied consent, (c) assured that their survey responses would be anonymous, (d) informed that they had the right not to answer any survey questions, and (e) informed that they had the ability to quit the survey at any time without penalty. Only participants who provided full informed consent participated in the survey. Participants who did not provide full informed consent were excluded and were unable to proceed with the survey. In addition, the participants were instructed to respond to all survey questions as honestly as they could, as the researchers were mainly interested in their opinions. Each survey took approximately 30 min to complete.

### 3.3 Materials

#### 3.3.1 Workplace incivility

Workplace incivility was measured using the Workplace Incivility Scale (WIS) [54]. The participants were asked to assess how often they had encountered specific rude or uncivil behaviors in their workplaces in the past year. The WIS has been widely used to measure workplace incivility [8,[96], [97], [98], [99]] and has demonstrated good psychometric properties [54]. The scale consisted of 12 items, such as “Gave you hostile looks, stares, or sneers?” Items were measured on a five-point Likert scale ranging from 1 (*Once or twice*) to 5 (*Many times*). Higher WIS scores reflect higher frequencies of workplace incivility. The WIS has high internal consistency, with an alpha of .89. The Cronbach's alpha for the WIS in the current study was 0.95, indicating good internal consistency.

#### 3.3.2 Serious organizational responses to complaints

This measure was modified from Hulin et al. and Martin and Hine's workplace incivility measure [99,100]. Specifically, this measure instructs participants to rate the likelihood of their workplace incivility complaints being taken seriously by their organizations. This measure consists of 13 items measured on a 4-point Likert scale ranging from 0 (*There is no chance that I would be taken seriously—0%*), 1 (*There is almost no chance that I would be taken seriously—25 %),* 2 (*There is a chance that I would be taken seriously—50 %*), and 3 (*There is a very good chance that I would be taken seriously—75 %*). An example item is “Repeatedly made insulting or disrespectful remarks about you.” Cronbach's alpha for the current sample was .94, indicating excellent internal consistency.

#### 3.3.3 Work satisfaction

An abridged job on a general scale was used to measure participants’ overall work satisfaction [66]. This scale measures global job satisfaction. This scale consists of five items, with two reverse-coded items. The responses ranged from 1 (*Strongly disagree*) to 5 (*Strongly agree*). An example item is, “I find real enjoyment in my work.” This scale has an excellent internal consistency of 0.83.

#### 3.3.4 Work withdrawal

Work withdrawal was measured using a modified 7-item version of the Work Withdrawal Measure [63]. Work withdrawal refers to the frequency of thoughts and behavioral engagements that dissatisfied employees take to avoid participating in their work [63]. Two example items include, “Completed work assignments late” and “Frequent/long coffee/lunch breaks,” with responses ranging from 1 (*Never*) to 8 (*Hourly*). The Cronbach's alpha for the current sample was. 89, indicating good internal consistency.

#### 3.3.5 Country of origin

We classified the country of origin of our participants using dummy coded variables, where a value of 1 was assigned to the Australian sample, and a value of 0 was assigned to the Singaporean sample.

#### 3.3.6 Gender

Gender was dummy coded, using a value of 1 for male and 0 for female.

### 3.4 Statistical analysis

This study used rigorous quantitative techniques to examine the scales and construct a path-based structural model (Partial least square structural equation modeling, PLS-SEM). Several steps were taken to reduce the bias and ensure accurate results. For example, we took care in preparing the questionnaire and in data processing, entry, cleaning, and analyses. To mitigate non-response bias, an inclusive sample representative of employees from various countries was included (see Table 1 for respondent profiles).

To establish reliable and consistent findings, measurements from relevant studies were adopted and systematic approaches, such as pre-testing, pilot testing, normality testing, exploratory factor analysis (EFA), and Cronbach's alpha reliability analysis, were employed. The Kaiser (Meyer) Olkin sampling adequacy measure score, which indicates the appropriateness of the sample for analysis, was exceptionally high at 93 %, surpassing the recommended threshold of 0.70 [101]. Multicollinearity issues that could result in biased coefficient estimations were also evaluated based on the Variance Inflation Factor (VIF) value [102]. In this study, a VIF greater than 10 was treated as indicative of multicollinearity in the factors or variables; therefore, variables were omitted to reduce the bias [102]. No evidence of multicollinearity was found in this study, as reported in the results section below. In addition, Fornell and Larker's matrix was used to measure convergent and divergent validity (i.e., AVE value) [103].

Furthermore, a data refining and screening process was carried out, which resulted in the removal of 13 of the 620 returned survey responses owing to a considerable amount of missing data, leaving a total of 607 viable responses for analysis. Because the items were collected from established scales, no confirmatory factor analysis (CFA) was conducted in the PLM-based SEM analysis. Instead, the model fit criterion and the values of R2, f2, and Q2 were used for a confirmatory composite analysis (CCA), an alternative method to a CFA in a PLS-SEM analysis [104]. Bootstrapping methods were used to strengthen the robustness of the PLS-SEM outcome and define statistical significance [104]. Finally, the moderating influence of demographic variables was examined using multigroup analysis (MGA) [105].

## 4 Results

### 4.1 Exploratory factor analysis, factor reliability, and factor validity

EFA was used to minimize the number of items within certain factors by considering their interrelationships. The scale items used in this study were interrelated and distinct from other groups of items. The selected items were generated from an established scale and further examined to analyze their interrelationships and assess the reliability of the scale. Items with values greater than or equal to 0.70 were considered acceptable for factor construction [101]. We found that 13 items were substantially interrelated and effectively measured the construct of serious organizational responses to complaints. Additionally, 12 items were found to be related to workplace incivility.

In addition, four scale items defined the “satisfaction” component, whereas five items defined the “work withdrawal” element. Furthermore, the Kaiser-Meyer-Olkin (KMO) value, which analyzes the appropriateness of sampling for factor analysis, considerably supported the dependability and utility of the obtained factors for creating the models (see Table 2). As presented in Table 2, all items had values greater than or equal to 0.70; therefore, the items are inter-related in defining the factors.

**Table 2:** Factor loading and significance.

Factors	Original sample (O)	Standard deviation (STDEV)	T statistics (|O/STDEV|)	P values	Cronbach's alpha	Composite reliability (Rho_c)
OTWI_Comp1 <- Seriousness complain	0.861	0.009	95.978	0.000	0.980	0.982
OTWI_Comp10 <- Seriousness complain	0.886	0.008	116.511	0.000		
OTWI_Comp11 <- Seriousness complain	0.924	0.005	177.364	0.000		
OTWI_Comp12 <- Seriousness complain	0.900	0.007	136.740	0.000		
OTWI_Comp13 <- Seriousness complain	0.929	0.005	176.168	0.000		
OTWI_Comp2 <- Seriousness complain	0.890	0.008	118.054	0.000		
OTWI_Comp3 <- Seriousness complain	0.811	0.012	66.104	0.000		
OTWI_Comp4 <- seriousness complain	0.895	0.008	118.148	0.000		
OTWI_Comp5 <- seriousness complain	0.887	0.007	122.341	0.000		
OTWI_Comp6 <- seriousness complain	0.935	0.005	193.471	0.000		
OTWI_Comp7 <- seriousness complain	0.938	0.004	227.412	0.000		
OTWI_Comp8 <- seriousness complain	0.901	0.007	137.853	0.000		
OTWI_Comp9 <- seriousness complain	0.914	0.006	156.416	0.000		
WIS_Inst1 <- Workplace Incivility	0.704	0.025	28.606	0.000	0.940	0.942
WIS_Inst10 <- Workplace Incivility	0.812	0.013	62.793	0.000		
WIS_Inst11 <- Workplace Incivility	0.839	0.011	73.455	0.000		
WIS_Inst12 <- Workplace Incivility	0.713	0.019	38.169	0.000		
WIS_Inst2 <- Workplace Incivility	0.752	0.022	34.742	0.000		
WIS_Inst3 <- Workplace Incivility	0.723	0.022	32.940	0.000		
WIS_Inst4 <- Workplace Incivility	0.812	0.012	65.498	0.000		
WIS_Inst5 <- Workplace Incivility	0.697	0.021	33.538	0.000		
WIS_Inst6 <- Workplace Incivility	0.766	0.016	46.932	0.000		
WIS_Inst7 <- Workplace Incivility	0.787	0.016	49.513	0.000		
WIS_Inst8 <- Workplace Incivility	0.854	0.008	105.296	0.000		
WIS_Inst9 <- Workplace Incivility	0.776	0.014	55.768	0.000		
Work_sat1 <- Work satisfaction	0.779	0.029	27.246	0.000	0.809	0.810
Work_sat2 <- Work satisfaction	0.853	0.013	63.802	0.000		
Work_sat4 <- Work satisfaction	0.792	0.021	38.370	0.000		
Work_sat5 <- Work satisfaction	0.539	0.040	13.335	0.000		
Work_with2 <- Work withdraw	0.736	0.021	35.244	0.000	0.845	0.849
Work_with3 <- Work withdraw	0.862	0.011	76.731	0.000		
Work_with4 <- Work withdraw	0.720	0.022	32.748	0.000		
Work_with5 <- Work withdraw	0.781	0.017	46.423	0.000		
Work_with6 <- Work withdraw	0.822	0.016	51.920	0.000		
KMO-	0.93					

Cronbach's alpha and composite reliability values were calculated to further explore reliability. A Cronbach's alpha greater than 0.70 and a composite reliability greater than 0.70 ensure that the items are reliable and consistently dependable in defining the factors. The Cronbach's alpha values for the constructs of seriousness of complaints, workplace incivility, satisfaction, and withdrawal were 0.98, 0.94, 0.73, and 9.85, respectively (see Table 2). Consequently, the reliability of the items in defining the elements was proven. Ensuring data dependability requires the absence of random errors. Random errors must be avoided to generalize the quantitative findings.

Once data dependability is established, validity must be established to ensure the absence of systematic mistakes. Three crucial measures were required to verify the validity of the model: content, convergent, and discriminant validity.

The content validity of this study was confirmed because the scale items used to define the factors were obtained from a previously established scale, and the content of the modified scales was checked by qualified researchers [106]. Convergent validity, which analyzes how items on a scale correlate with one another to construct a certain factor, uses two essential metrics for establishing validity: composite reliability and average variance extracted (AVE) [101]. We found that the composite reliability exceeded the 0.70 level (see Table 2) and the AVE was greater than 0.50 (see Table 3), confirming the convergent validity of the scales.

**Table 3:** Discriminate validity.

Validity: Fornell-Larcker criterion					
	Average variance extracted (AVE)	Complain	seriousness complains	Work satisfaction	Work Withdrawal
Workplace Incivility	0.604	0.771			
seriousness complains	0.807	−0.216	0.899		
Work satisfaction	0.724	−0.263	0.200	0.750	
work withdraw	0.619	0.254	0.205	−0.219	0.786

Importantly, discriminant validity is obtained when the square root value of each AVE is greater than the correlation between all pairs of latent constructs [107]. The Fornell–Larcker criterion was used to analyze the square root value of each AVE and the correlation of all pairings of latent constructs. The Fornell-Larcker criterion matrix's on-diagonal elements reflect the square root value of each AVE, while its off-diagonal elements indicate the correlations. Based on the data in Table 3, the discriminant validity of the scale items was verified. Overall, the above results for reliability and validity confirmed that the constructs were valid for the development of the model.

### 4.2 PLS model evaluation and model building

PLS-SEM models were constructed based on the following wagers: workplace incivility negatively affects work satisfaction (H1); workplace incivility positively influences work withdrawal (H2); work withdrawal negatively affects work satisfaction (H3); the negative relationships between workplace incivility and work satisfaction are stronger when organizations do not take complaints seriously than when they do (H4); and the positive relationships between workplace incivility and work withdrawal are stronger when organizations do not take complaints seriously than when they do (H5).

**Fig. 2:**
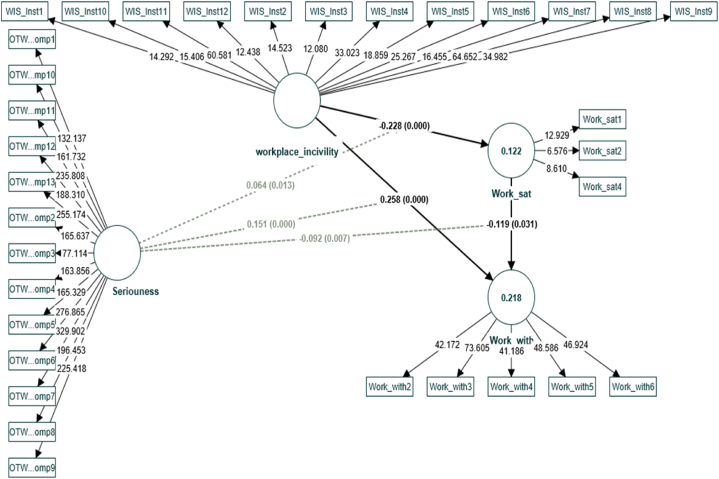
Path model.

Initially, a basic path model was drawn to investigate the model fit indices and path strength based on the CCA [104]. The model fit indices outlined in Table 4 confirmed that the proposed PLS-SEM achieves a satisfactory model fit. We found that the SRMR value was less than 0.10, the NFI values were closer to 1, the RMS theta values were less than 12, and the VIF values were less than 10. The VIF value indicated that the models were free of multi-collinearity. The theta showed that there were fewer residuals in the outer models, the SRMR confirmed that the average differences between the observed and expected correlations were small, and the NIF confirmed that the net fit indices were reasonable for accepting the model. Consequently, the path model outlined in Fig. 2 was built.

**Table 4:** PLS Model fit Indices.

Criterion	Meaning	References	Referred value	Referred value	Estimated Model	Saturated Model	Model evaluation
SRMR (Standardized Root Mean Square Residual)	Difference between the observed correlation and the model implied correlation matrix	Hu and Bentler, (1999)	<0.10	<0.10	0.09	0.09	Accepted
Chi-Square		Lohmöller (1989)	No referred value	No referred value	7341	7341	
NFI (Normed Fit Index)	Incremental fit measure	Bentler and Bonett (1980)	closer the NFI to 1, the better the fit	closer the NFI to 1, the better the fit	0.83	0.83	Reasonable Accepted
rms Theta	Degree to which the outer model residuals correlate.	Henseler et al. (2014)	<12	<12	0.11	0.11	Accepted
VIF (Variance Inflation Factor)	Measures the severity of multicollinearity in regression analysis	(Hair et al., 1995); (Ringle et al., 2015)	<10 or < 5	<10 or < 5	<2	<2	Accepted

Furthermore, the *R*^2^ and *f*^2^ values were investigated [104]. As suggested by, *R*^2^ and *f*^2^ values > 0.15 indicate that the effect of the path model is small but may be significant in defining the path relationship. Standard bootstrapping procedures were used with 5000 resamples to further corroborate the path relationship. Table 5 shows the results of the PLS bootstrapping approaches and Table 6 illustrates the variations in the paths based on gender, country of origin, and combinations.

**Table 5:** Hypothesis testing.

	Concern	Measures	Work Incivility- > Work satisfaction	Work Incivility - > Work withdrawal	Work withdrawal - > Work satisfaction	Complain taken seriously x Work Incivility - > Work satisfaction	Complain taken seriously x work incivility - > Work withdrawal	Accept/Reject
H1	Combined Model	Coefficient	−0.15					Accepted
		p values	0.00					
H2		Coefficient		0.33				Accepted
		p values		0.00				
H3		Coefficient			−0.23			Accepted
		p values			0.00			
H4		Coefficient				0.09		Accepted
		p values				0.04		
H5		Coefficient					0.16	Accepted
		p values					0.00	
H6	Australia	Coefficient	−0.18	0.41		−0.05	0.24	Accepted
		p values	0.00	0.00		0.36	0.00	
	Singapore	Coefficient	−0.11	0.43		0.17	0.15	
		p values	0.10	0.00		0.00	0.01	
H7	Australian Female	Coefficient	−0.16	0.54		0.12	0.09	Accepted
		p values	0.12	0.00		0.26	0.44	
	Australian Male	Coefficient	−0.29	0.28		−0.11	0.27	
		p values	0.00	0.00		0.14	0.00	
	Singapore female	Coefficient	−0.06	0.43		0.18	0.20	
		p values	0.61	0.00		0.05	0.00	
	Singapore male	Coefficient	−0.16	0.42		0.17	0.11	
		p values	0.10	0.00		0.03	0.27

**Table 6:** Multi-group mean differences.

*Paths*	*Female vs Male*		*Australian Female vs male*		*Australia vs Singapore*		*Singapore Female vs male*		*Australian Female vs Singapore female*		*Australian male vs Singapore male*	
	Difference	2-tailed- p value	Difference	2-tailed- p value	Difference	2-tailed- p value	Difference	2-tailed- p value	2-tailed- p value	Difference	2-tailed- p value	Difference
H1: Work Incivility- > Work satisfaction	0.02	0.00	0.16	0.00	−0.07	0.00	0.11	0.00	−0.11	0.00	−0.16	0.00
H2: Work Incivility - > work withdrawal	0.20	0.02	0.27	0.00	−0.02	0.00	0.02	0.00	0.13	0.00	−0.16	0.00
H4: Seriousness complains x work incivility - > Work satisfaction	0.01	0.00	0.26	0.00	−0.24	0.00	0.03	0.00	0.06	0.00	−0.30	0.00
H5: Seriousness complains x Work incivility - > work withdraw	0.02	0.79	−0.21	0.00	0.10	0.00	0.08	0.00	−0.12	0.00	0.17	0.00

The results supported all the hypothesized relationships (see Table 5). We found that workplace incivility negatively influenced work satisfaction (H1) (*β* = −0.15, *p* < 0.01) and positively influenced work withdrawal (H2) (*β* = 0.33, *p* < 0.01). Furthermore, work withdrawal negatively affected work satisfaction (H3) (*β* = −0.23, *p* < 0.01). These results reveal that workplace incivility is a serious issue in the organizational realm: it hinders employee satisfaction and encourages work withdrawal; in turn, work withdrawal further worsens satisfaction.

In the case of H4, we found that serious organizational responses to work incivility complaints moderated the relationships between workplace incivility and work satisfaction (H4) (*β* = 0.09, *p* < 0.05). This finding indicates that the negative impact of workplace incivility can be mitigated when organizations take incivility-related concerns seriously; that is, when organizations treat workplace incivility as a serious issue, employees are more likely to be satisfied at work. Thus, serious organizational responses to workplace incivility complaints might assist employees in discovering a sense of belonging, which may enhance their work satisfaction.

In the case of H5, the findings showed that serious organizational responses to incivility complaints moderated the relationship between workplace incivility and work withdrawal (H5) (*β* = 0.16, *p* < 0.01). This interesting finding may suggest that while serious organizational responses to work incivility complaints can lessen work withdrawal (*β* decreased from 0.33 to 0.16), it may not eliminate it. This may be because, even when complaints are taken seriously, employees may still have negative associations with the initial circumstances that triggered the complaint. For instance, if an employee complains about the behavior of a colleague and the matter is resolved, the employee may still feel uncomfortable around that co-worker or have negative feelings toward them. Furthermore, the process of filing a complaint and resolving it can be unpleasant and time-consuming for employees. Thus, even if complaints are eventually handled to support employee satisfaction, the stress and distraction associated with workplace incivility might contribute to work withdrawal.

The above findings underline the relevance of employers listening to and reacting swiftly to employee complaints to foster work satisfaction and lower work withdrawal rates. Moreover, resolving the underlying issues that cause workplace incivility would be beneficial to organizations. Next, we investigated the impact of country context and gender on the relationships between complaints, work satisfaction, withdrawal, and complaint severity.

### 4.3 Multigroup analysis (country of origin, hypothesis 6)

H6 posited that Australian participants will be more adversely affected by work incivility (via work withdrawal and work satisfaction) than Singaporean participants. We identified notable differences between Australia and Singapore regarding the relationship between workplace incivility and work-related outcomes. In Australia, we observed a significant negative relationship between workplace incivility and work satisfaction (*β* = −0.18, *p* < 0.01) and a significant positive relationship between workplace incivility and work withdrawal (*β* = 0.41, *p* < 0.01). Conversely, in Singapore, we observed a significant positive relationship between workplace incivility and work withdrawal (*β* = 0.43, *p* < 0.01), but did not observe any significant relationship between incivility and work satisfaction (*β* = −0.11, *p* > 0.05). Comparing the work contexts of Australia and Singapore, we can conclude that workplace incivility has a more severe effect on work dissatisfaction in Australia than in Singapore because it significantly reduces work satisfaction and increases work withdrawal in Australia.

Regarding the moderating effect of serious organizational responses to complaints, we observed that when incivility complaints are taken seriously in Australia, work withdrawal significantly declines (*β* decreases from 0.41 to 0.24); however, interestingly, the degree to which organizations take incivility complaints seriously had no significant effect on work satisfaction (*β* = −0.05, *p* > 0.05). Conversely, in Singapore, the degree to which organizations take incivility complaints seriously significantly mitigated the relationship between work incivility and work withdrawal (*β* decreased from 0.43 to 0.15, *p* < 0.01) and increased work satisfaction (from −0.11 to 0.17, *p* < 0.01) among Singaporean employees.

This result implies that the degree to which incivility complaints are taken seriously does not improve satisfaction but can decrease work withdrawal among Australian workers. This finding may reflect that Australian employees perceive incivility as an extremely serious issue when lodging complaints. Since Australian organizations that take incivility complaints seriously are less likely to increase their workers’ satisfaction and more likely to minimize job withdrawal when incivility occurs, proactive tactics are required in Australia to eliminate incivility and manage personnel.

These results highlight the influence of cultural differences in the complex relationships between workplace incivility and its outcomes. While workplace incivility was not significantly related to work satisfaction in Singapore, it had a strong negative impact on satisfaction in Australia. Additionally, contrary to the findings in Australia, serious organizational responses to complaints in Singapore tended to have a positive impact on work satisfaction. Cultural factors, such as the value placed on harmony or the relevance of authoritative figures in mediating disputes, may explain these variations.

### 4.4 Multigroup analysis (gender)

Our analysis of the PLS models (Table 5, Table 6) indicated that Australian males exhibit greater sensitivity (*p* < 0.001) to workplace incivility—evidenced through their related work satisfaction and work withdrawal—compared to Australian females (*p* > 0.05), Singaporean males (*p* > 0.05), and Singaporean females (*p* > 0.05). Put differently, we found that workplace incivility most negatively impacts work satisfaction and most intensely increases the likelihood of withdrawing from work among Australian males. Interestingly, serious organizational responses to work incivility complaints did not significantly moderate the effects of work incivility on the work satisfaction or work withdrawal intentions of Australian males (*p* > 0.05). The withdrawal effect remained almost the same (*β* = 0.27) regardless of the moderator of how seriously organizations responded to incivility complaints. This outcome supports our assumption that Australian male participants are likely to experience more adverse impacts in terms of work withdrawal and work satisfaction than both Australian female participants and Singaporean participants, even when complaints are taken seriously by their organizations. In summary, the above findings demonstrate that both country context and gender significantly influenced the path diagram, supporting our hypotheses.

## 5 Discussion

In this cross-cultural study, we tested a moderated model to determine whether the moderator (i.e., serious organizational responses to victims' complaints) influences the relationship between workplace incivility (e.g., independent variable) and employees' organizational outcomes (e.g., work satisfaction and withdrawal) in a sample of Australian and Singaporean white-collar workers. H1 and H2 proposed that workplace incivility is negatively related to work satisfaction and positively related to work withdrawal; both H1 and H2 were fully supported. These results make sense: workplace incivility likely depletes victims’ mental resources, leading to heightened stress and negative organizational outcomes, such as work dissatisfaction and withdrawal [29,65]. In addition, the results indicated that employee withdrawal reflects their dissatisfaction with their current work [63,108]; this finding fully supported H3.

H4 and H5 predicted that the degree to which organizations take seriously the complaints of victims of workplace incivility would moderate the relationship between negative work experiences (e.g., workplace incivility) and organizational outcomes. For instance, when organizations fail to seriously consider victims' workplace incivility complaints, they can influence their employees' sense of organizational justice, which can, in turn, decrease their employees’ work satisfaction and increase their work withdrawal. The findings of our analysis supported H4 and H5.

Next, we combined the influence of two macro-level context variables (e.g., national/cultural/country of origin and gender) with organizational responses to victims' complaints to determine how they influenced victims’ organizational outcome variables (i.e., work satisfaction and withdrawal). H6 argued that the experiences and identities of victims of workplace incivility are colored by their exposure to overlapping forms of cultural and societal discrimination and privileges. For instance, victims from high power distance cultures (e.g., Singapore) are less affected by their organizations ignoring their incivility complains than victims from low power distance cultures because they are conscious of the need to defer to their supervisors, maintain group harmony, and prevent trouble for their group members [82]. The results of our study fully supported H6. However, we would also like to point out that the ways in which organizations manage victim complaints are influenced by the existence or absence of existing policies and procedures [90]—not just cultural factors.

Similarly, gender is likely to influence the experiences of female victims from different cultures. For instance, researchers have found that female academic experiences are influenced by perceptions of sexism [109]. Similarly, compared to male victims of workplace incivility, female targets were less able to withdraw from their work because of gendered societal norms which compelled them to be more accepting and tolerant of injustices [92]. In the context of the current study, this existing work suggests that female workers in both Australia and Singapore are less likely to be adversely affected by workplace incivility than male victims in these countries, even when their organizations take their incivility complaints seriously; this argument was captured in H7. Our study found that Australian males are most affected by workplace incivility; thus, H7 was confirmed.

### 5.1 Limitations

This study had some limitations. First, its cross-sectional nature prevented us from inferences beyond its tenuous sample. Future researchers could consider analyses that compare organizational responses to victims’ complaints using a longitudinal approach to determine whether significant changes will occur over time. Nevertheless, this study provides preliminary findings about the cross-cultural experiences of targets of workplace incivility and how they respond when their complaints are ignored or taken lightly by their organizations. However, given the ambiguous nature of workplace incivility, future studies should consider incorporating a qualitative component to determine whether targets of workplace incivility differently perceive the ambiguous nature and/or intentions of perpetrators of workplace incivility. This is important because cross-cultural studies have found that perception and tolerance towards organizational injustice and mistreatment vary according to national power distances [[29], [30], [31]]. Moreover, future research should consider replicating this study in developing countries, where there are looser regulations for workplace misconduct and organizational responses to employee mistreatment in the workplace. Finally, we were unable to fully investigate the potential effects of other demographic variables as they were outside of our proposed models. However, researchers interested in this topic may wish to include other demographic variables (e.g., emotional intelligence) when investigating workplace incivility among diverse groups.

### 5.2 Theoretical implications

The results of this study have several theoretical and practical implications. First, we notably adopted an ecological approach, which expanded the analysis of workplace incivility beyond the individual. Second, in examining cross-cultural responses to workplace incivility among Australian and Singaporean employees, we offered preliminary insights into how diverse cultural contexts shape individual responses to workplace incivility. Finally, we uncovered the moderating role of the degree to which organizations take targets of workplace incivility complaints seriously on the workplace incivility–outcome nexus across cultures and genders. In sum, this study offers important insights into cross-cultural variations in workplace incivility and the impact of organizational responses to incivility that organizations can apply to manage its adverse effects across diverse contexts.

### 5.3 Practical implications

Our findings have important practical implications for HR leaders, policymakers, and managers. Many organizations have anti-bullying policies or dignity/respect in the workplace policies. While these policies are good, they alone are insufficient to deal with the complex and problematic nature of workplace incivility. Specifically, power differentials exist between the target and the perpetrator as well as at the organizational and societal levels. For example, at the micro level, many managers choose to ignore workplace incivility because they do not want to deal with complex interpersonal conflicts. However, power differentials also exist in organizations and societies. Compared to men, women generally form less powerful social and professional networks; this tendency isolates women from support networks that may buffer against stress [110]. Consequently, women tend to internalize the occurrence of incivility or harassment and blame themselves. Researchers have found that unlike their male counterparts, women who encountered incivility in their workplaces were less willing to “rock the boat” or withdraw from their work [92]. From an organizational perspective, establishment structures also play a vital role in the perception of justice. For instance, victims who made formal complaints but were ignored by their organizations consequently felt that they were not valued in their society [87]; this sense of having a low societal value can, in turn, cause victims to feel that they are invisible, unworthy, and excluded from their organizations [111].

Given the complex ways in which workplace incivility is embedded across the micro- and macro-levels, organizations should implement multidimensional interventions or strategies to tackle workplace incivility. Broadly, organizations should commit to promoting civility in all exchanges. They can do this by introducing formal anti-bullying and/or respect in the workplace policies and by requiring senior employees to exemplify good workplace behaviors. Ultimately, supervisors with line authority must take their management responsibilities seriously and avoid ignoring or neglecting staff complaints to normalize appropriate workplace behavior. In addition, by seriously attending to staff complaints, organizations can restore employee perceptions of organizational justice, which has been shown to impact employee performance, well-being, and interpersonal relationships [112,113].

It is also important that managers, HR leaders, and staff are all provided with appropriate training on acceptable and unacceptable workplace behaviors, including appropriate procedures for resolving such conflicts [114]. Many managers are ignorant of workplace incivility or perceive such conflicts to be interpersonal and unworthy of their attention. Managers should take victim complaints seriously and accordingly intervene in such conflicts early on to curb their consequences. In addition, managers should convey to all staff that their complaints will be handled seriously, confidentially, and effectively in a transparent manner. This is important because workplace incivility, if left unattended, can escalate into violent behavior [29]. By taking these steps, managers can show staff that they respect them as valued members of their organization; therefore, these actions can empower staff and reduce their stress. Given that experiencing workplace incivility is a stressor, organizations should also implement mindfulness intervention programs, such as workshops for stress coping strategies and emotional regulation, which are highly effective in reducing emotional exhaustion and improving job satisfaction [115,116]. Finally, it is vital that organizations develop and implement formal and informal policies and procedures to manage target's complaints which can include structured reporting mechanisms, educational workshops, and/or counselling sessions such as stress coping strategies and mindfulness training, so as to readily address and prevent workplace incivility [117].

## 6 Conclusion

This study demonstrated that organizations should effectively handle target incivility complaints because their responses directly impact their organizational outcomes. Moreover, in addressing such complaints, managers and HDR leaders should be aware of the impact of cultural and gendered factors on victims’ perceptions of fairness. Accordingly, to maintain a healthy workplace environment and ensure employee well-being, organizations should take victim complaints seriously and consider the cultural and gendered dimensions of incivility; for example, these approaches are important when implementing policies for anti-bullying and respect in the workplace and addressing victim compliance.

## Ethical declarations

All protocols for this study were reviewed and approved by the Human Research Ethics Committee of Edith Cowan University (approval number:10230LOH).

## Consent

All participants provided informed consent to participate in the study through the completion of the survey questionnaire.

## Funding

This research was funded by the Faculty of Health, Engineering and Science (Collaborative Research Grant, 10.13039/501100001798Edith Cowan University (Australia).

## Data availability statement

Data has not been deposited into a publicly available repository. Data will be made available on request.

## Additional information

No additional information is available for this paper.

## CRediT authorship contribution statement

**Jennifer (M.I) Loh:** Conceptualization, Data curation, Formal analysis, Funding acquisition, Investigation, Methodology, Project administration, Resources, Validation, Writing – original draft, Writing – review & editing. **Md Irfanuzzaman Khan:** Formal analysis, Methodology, Validation, Writing – original draft, Writing – review & editing. **MdJakir Hasan Talukder:** Formal analysis, Methodology.

## Declaration of competing interest

The authors declare that they have no known competing financial interests or personal relationships that could have appeared to influence the work reported in this paper.
