# Supplementary material for: To complain or not to complain: Management responses as a moderator in the relationship between workplace incivility and workplace outcomes among Australia and singaporean targets
Source: Heliyon. 2023 Oct 21;9(11):e21363. doi: 10.1016/j.heliyon.2023.e21363 (PMC10613909; doi:10.1016/j.heliyon.2023.e21363)
Supplement: Multimedia component 1 [file mmc1.docx]

**Questionnaire**

**General Instructions**

This research study has been designed to identify beliefs, behaviours, and workplace conditions relating to well-being in the workplace. This survey will take approximately 15-20 minutes and we will appreciate your participation. Please bear in mind that there is **NO** right or wrong answer as we are primarily interested in your opinion. Therefore, please be as honest and accurate as you can throughout the survey. Your responses on this survey will remain anonymous.

**Personal Demographic Information**

(Please provide us with some information about yourself. We will use this information only to describe in general terms the group of people who completed the survey)

- What is your gender?
- Male
- Female
- What is your age? ____(in years)
- Are you:
- Married/Defacto
- Single
- Separated
- Divorced
- Widowed/Widower
- Do you have any children? If yes, please indicate number of children? ____
- What is your highest education attainment to date?
- Year 10 or below
- Year 12
- TAFE certificate/diploma
- University degree (i.e., Bachelor degree)
- Higher degree (i.e., Masters, PhD)
- Other:________________________
- What type of work have you done in the last 12 months _____________? Has the work been: Full time, Part time, or Casual?
- If you do work, please indicate on average the numbers of hours you work per week? _______
- Please indicate your country of origin _____________________
- Which of the following best describes the industry you work in?
  - Banking
  - Insurance
  - Agriculture, Forestry, & Fishing
  - Mining
  - Manufacturing
  - Electricity, Gas, & Water Supply
  - Construction
  - Wholesale Trade
  - Retail Trade
  - Accommodation, Cafes, & Restaurants
  - Transport & Storage
  - Communication Services
  - Property & Business Services
  - Government Administration
  - Defence
  - Education
  - Health & Community Services
  - Cultural & Recreational Services
  - Personal & Other Services
  - Other (Please Specify Below)

If “Other”-please specify your industry _______________________

- Please indicate the length (in years and months) you have been employed in your current company? ________
- How much did you earn in the previous financial year:
  - 0 - $18,200
  - $18,201 - $37,000
  - $37, 001-$54,000
  - $54,001-$72,800
  - $72,801-$91,600
  - Above $91,601

**Instructions:** Please indicate how often you have **experienced** the following behaviours in the past year where (0= Never, 1= Once or twice, 2= Sometimes, 3= Often and 4=Many times).

How often have you experienced the following behaviours in the past year from this person/s?

1. Paid little attention to your statements or showed little interest in your opinions.
2. Doubted your judgment in a matter over which you have responsibility.
3. Gave you hostile looks, stares, or sneers.
4. Addressed you in unprofessional terms, either privately or publicly.
5. Interrupted or “spoke over” you.
6. Rated you lower than you deserved on an evaluation.
7. Yelled, shouted, or swore at you.
8. Made insulting or disrespectful remarks about you.
9. Ignored or failed to speak to you (e.g., gave you the “silent treatment”)
10. Accused you of incompetence.
11. Targeted you with anger outbursts or “temper tantrums”.
12. Made jokes at your expense.

**Modified Workplace Incivility Scale**

|  |
| --- |
| **Instructions:** Please indicate how strongly you agree or disagree with each of the following statement (Please circle your answer where 1 = Strongly Disagree and 5= Strongly Agree).   1. I feel fairly well satisfied with my present job 2. Most days I am enthusiastic about my work 3. Each day of work seems like it will never end* 4. I find real enjoyment in my work 5. I consider my job rather unpleasant *   **Work satisfaction**  ***Reverse score**  **Instructions:** Please indicate how often you have **engaged** in the following behaviours where: (0= Never, 1= Once or twice a year, 2= once or twice every six months, 3= once or twice every month, 4=once or twice every week, 5= once or twice daily, 6= more than twice a day and 7= hourly).   1. Completed work assignments late. 2. Frequent/long coffee/lunch breaks. 3. Made excuses to get out of the office. 4. Been late for work. 5. Absent from work. 6. Neglected tasks not affecting evaluation/pay raise. 7. Thought about resigning from your current job.   ***Work Withdrawal*** |

**Instruction:** If you make a formal complaint against the perpetrator who engaged in the following behaviours, what (in your opinion) is the likelihood of the complaint **being taken seriously by your management**?

**0** (There is no chance that I would be taken seriously- 0%)

**1** (There is almost no chance that I would be taken seriously-25%)

**2** (There is a chance that I would be taken seriously-50%)

**3** (There is a very good chance that I would be taken seriously-75%)

1. Repeatedly treated you in a hostile manner.
2. Repeatedly invaded your privacy.
3. Repeatedly gossiped about you to co-workers.
4. Regularly withheld important information relevant to your job and/or excluded you from key decisions.
5. Repeatedly disregard your opinions or judgement in matters over which you have responsibility.
6. Repeatedly made insulting or disrespectful remarks about you.
7. Repeatedly addressed you in unprofessional terms, either privately or publicly.
8. Repeatedly interrupted or “spoke over” you.
9. Repeatedly yelled, shouted, or swore at you.
10. Repeatedly ignored or failed to speak to you (e.g., gave you the “silent treatment”
11. Repeatedly accused you of incompetence.
12. Repeatedly targeted you with anger outbursts or “temper tantrums”.
13. Repeatedly made jokes at your expense.

**Modified Organisational Tolerance for Workplace Incivility**
